# SciSciNet: A large-scale open data lake for the science of science research

**DOI:** 10.1038/s41597-023-02198-9

**Published:** 2023-06-01

**Authors:** Zihang Lin, Yian Yin, Lu Liu, Dashun Wang

**Affiliations:** 1 Center for Science of Science and Innovation, Northwestern University, Evanston, IL USA; 2 Northwestern Institute on Complex Systems, Northwestern University, Evanston, IL USA; 3 Kellogg School of Management, Northwestern University, Evanston, IL USA; 4 School of Computer Science, Fudan University, Shanghai, China; 5 McCormick School of Engineering, Northwestern University, Evanston, IL USA

**Keywords:** Scientific community, Publishing

## Abstract

The science of science has attracted growing research interests, partly due to the increasing availability of large-scale datasets capturing the innerworkings of science. These datasets, and the numerous linkages among them, enable researchers to ask a range of fascinating questions about how science works and where innovation occurs. Yet as datasets grow, it becomes increasingly difficult to track available sources and linkages across datasets. Here we present SciSciNet, a large-scale open data lake for the science of science research, covering over 134M scientific publications and millions of external linkages to funding and public uses. We offer detailed documentation of pre-processing steps and analytical choices in constructing the data lake. We further supplement the data lake by computing frequently used measures in the literature, illustrating how researchers may contribute collectively to enriching the data lake. Overall, this data lake serves as an initial but useful resource for the field, by lowering the barrier to entry, reducing duplication of efforts in data processing and measurements, improving the robustness and replicability of empirical claims, and broadening the diversity and representation of ideas in the field.

## Background & Summary

Modern databases capturing the innerworkings of science have been growing exponentially over the past decades, offering new opportunities to study scientific production and use at larger scales and finer resolution than previously possible. Fuelled in part by the increasing availability of large-scale datasets, the science of science community turns scientific methods on science itself^1–6^, helping us understand in a quantitative fashion a range of important questions that are central to scientific progress—and of great interest to scientists themselves—from the evolution of individual scientific careers^7–18^ to collaborations^19–25^ and science institutions^26–28^ to the evolution of science^2,3,5,29–34^ to the nature of scientific progress and impact^35^–^55^.

Scholarly big data have flourished over the past decade, with several large-scale initiatives providing researchers free access to data. For example, CiteSeerX^56^, one of the earliest digital library search engines, offers a large-scale scientific library focusing on the literature in computer and information science. Building on a series of advanced data mining techniques, AMiner^57^ indexes and integrates a wide range of data about academic social networks^58^. Crossref (https://www.crossref.org/)^59^, as well as other initiatives in the open metadata community, have collected metadata such as Digital Object Identifier (DOI) in each publication record and linked them to a broad body of event data covering scholarly discussions. OpenAlex (https://openalex.org/)^60^, based on Microsoft Academic Graph (MAG)^61–63^, aims to build a large-scale open catalog for the global research system, incorporating scholarly entities and their connections across multiple datasets. In addition to data on scientific publications and citations capturing within-science dynamics, researchers have also tracked interactions between science and other socioeconomic spheres by tracing, for example, how science is referenced in patented inventions^64–66^, regarding both front-page and in-text citations from patents to publications^67,68^. Table 1 summarizes several exemplary datasets commonly used in the science of science literature, with information on their coverage and accessibility.

**Table 1:** Brief summary of major data sources commonly used in the science of science literature.

Data source	Highlights	API	Data dump
Crossref	Data on publications with DOIs registered in Crossref.	✓	✓
OpenAlex	Data connecting publications, authors, institutions, and concepts.	✓	✓
Dimensions	Data connecting publications, grants, datasets, trials, and patents.	—	—
Overton	Policy documents and their citations to science and policy.	—	—
OpenCitations	DOI-DOI open citation links.	✓	✓
AMiner	Advanced information generated through data mining techniques.	✓	✓
CiteSeerX	Full-text publications, one of the earliest digital library search engines.	✓	—
ORCID	Data on researchers with ORCID IDs (funding, works, peer review, etc.).	✓	✓
ROR	Data on research organizations with ROR IDs, seeded by GRID.	✓	✓
Retraction Watch	Data on retracted papers and reasons for retraction.	✗	—
Semantic Scholar	Publication dataset featuring AI-derived products (e.g., embeddings).	✓	—
Web of Science	Curated by in-house experts, basis for Journal Citation Reports.	—	—
PubMed	Biomedical literature with PubMed IDs, linked to NIH projects, clinical trials, and other biomedical entities.	✓	✓
NIH RePORTER	Data on NIH-funded projects, with linkages to publications, patents, and clinical studies.	✓	✓
NSF Awards	Data on NSF-funded projects, with linkages to publications.	✓	✓
Clinical Trials	Information on clinical studies and linkages to references worldwide.	✓	✓
PatentsView	Data on USPTO patents (citations, classifications, inventors, etc.).	✓	✓
Patent Citation to Science	Patent-science citations extracted from USPTO and EPO patents.	✗	✓
Publications of Nobel laureates	Publication records and prize-winning papers of Nobel laureates.	✗	✓
Altmetric	Data on online attention (e.g., mainstream and social media).	✓	—
CORE	Metadata and full-text information of 87 M + papers.	✓	✓
Unpaywall	Publication metadata and open-access related information.	✓	✓
DOAJ	Community-curated data on open-access journals and papers.	✓	✓
OpenAIRE Research Graph	Data connecting scientific products, organizations, funded projects, etc. from 70 K + sources.	✓	✓
Faculty Opinions with Gender	Metadata of authors from Faculty Opinions with gender classification from Faculty Opinions and Web of Science.	—	✓
Scopus	Documents selected by an independent review board of experts.	—	—
Lens	Citation relationships within and across papers and patents.	—	—
Springer Nature SciGraph	Triples connecting multiple entities in the research landscape, including publications, funders, and affiliations.	✓	✓
Google Scholar	Large-scale data on publications, citations, and disambiguated scholar profiles indexed by Google.	✗	✗

✓: publicly available, —: available upon application or subscription, ✗: not available to the best of our knowledge (a more detailed summary is given in Table S1).

The rapid growth of the science of science community^69–71^, combined with its interdisciplinary nature, raises several key challenges confronting researchers in the field. First, it becomes increasingly difficult to keep track of available datasets and their potential linkages across disparate sources, raising the question of whether there are research questions that are underexplored simply due to a lack of awareness of the data. Second, as data and their linkages become more complex, there are substantial data pre-processing steps involved prior to analyses. Many of these steps are often too detailed to document in publications, with researchers making their own analytical choices when processing the data. Third, as tools and techniques used in the science of science grow in sophistication, measurements on these datasets can be computationally involved, requiring substantial investment of time and resources to compute these measures.

All these challenges highlight the need for a common data resource designed for research purposes, which could benefit the community in several important ways. First, it provides a large-scale empirical basis for research, helping to strengthen the level of evidence supporting new findings as well as increase the replicability and robustness of these findings. Second, it helps to reduce duplication of efforts across the community in data preprocessing and common measurements. Third, by compiling various datasets, linkages, and measurements, the data resource significantly lowers the barrier to entry, hence has the potential to broaden the diversity and representation of new ideas in the field.

**Fig. 1:**
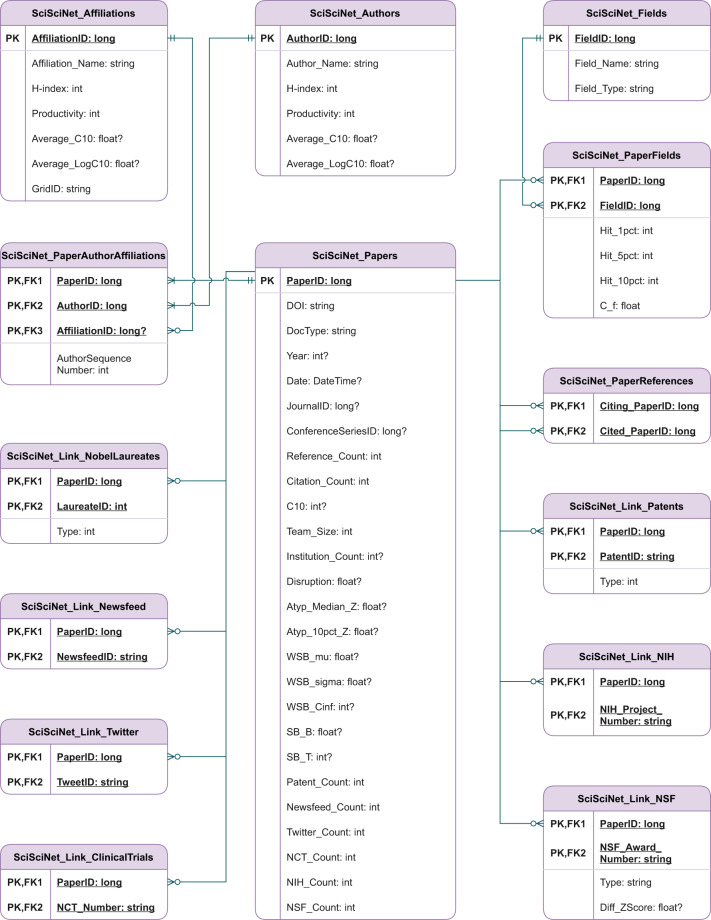
The entity relationship diagram of SciSciNet. SciSciNet includes “SciSciNet_Papers” as the main data table, with linkages to other tables capturing data from a range of sources. For clarity, here we show a subset of the tables (see Data Records section for a more comprehensive view of the tables). PK represents primary key, and FK represents foreign key.

To support these needs in the community, we present SciSciNet, a large-scale open data lake for the science of science research. The data lake not only incorporates databases that capture scientific publications, researchers, and institutions, but also tracks their linkages to related entities, ranging from upstream funding sources like NIH and NSF to downstream public uses, including references of scientific publications in patents, clinical trials, and media and social media mentions (see Fig. 1 and Table 2 for more details of entities and their relationships). Building on this collection of linked databases, we further calculate a series of commonly used measurements in the science of science, providing benchmark measures to facilitate further investigations while illustrating how researchers can further contribute collectively to the data lake. Finally, we validate the data lake using multiple approaches, including internal data validation, cross-database verification, as well as reproducing canonical results in the literature.

**Table 2:** Dataset descriptions.

File	Lines	Short Description (all files are in TSV format)
SciSciNet_Papers	134,129,188	File containing primary papers with Paper IDs, categories, counts, and calculated foundational metrics.
SciSciNet_PaperAuthorAffiliations	413,869,501	File containing paper-author-affiliation linkages.
SciSciNet_PaperReferences	1,588,739,703	File containing paper reference pairs within primary papers that appear in SciSciNet_Papers.
SciSciNet_Fields	311	File containing Field IDs with names and types (top-level or sub-level).
SciSciNet_Journals	49066	File containing Journal IDs with names, ISSNs, publishers, and official webpages.
SciSciNet_ConferenceSeries	4551	File containing Conference Series IDs with names.
SciSciNet_Authors_Gender	134,197,162	File containing Author IDs with names and individual career-level metrics.
SciSciNet_PaperFields	277,494,994	File containing linkages between Paper ID and Field ID.
SciSciNet_PaperDetails	136,726,948	File containing detailed information of papers (covering retracted papers and affiliated papers in paper families as well) including titles, journals, and publishers.
SciSciNet_Affiliations	26,998	File containing Affiliation IDs with names and institution-level metrics.
SciSciNet_Link_NSF	1,309,518	File containing linkages between Paper ID and NSF Award Number.
SciSciNet_Link_NIH	6,013,187	File containing linkages between Paper ID and NIH Project Number.
SciSciNet_Link_ClinicalTrials	438,220	File containing linkages between referenced Paper ID and NCT Number.
SciSciNet_Link_NobelLaureates	87,316	File containing linkages between Paper ID and Nobel Laureate ID.
SciSciNet_Link_Twitter	55,846,550	File containing linkages between Paper ID and Tweet ID.
SciSciNet_Link_Newsfeed	595,241	File containing linkages between Paper ID and Newsfeed ID.
SciSciNet_Link_Patents	38,740,313	File containing linkages between Paper ID and Patent ID.
SciSciNet_NSF_Metadata	489,446	File containing metadata of NSF awards from nsf.gov.
SciSciNet_Newsfeed_Metadata	947,160	File containing metadata of scientific mentions in Newsfeed from Crossref Event API.
SciSciNet_Twitter_Metadata	59,593,281	File containing metadata of scientific mentions in Twitter from Crossref Event API.

The data lake, SciSciNet, is freely available at Figshare^72^. At the core of the data lake is the Microsoft Academic Graph (MAG) dataset^61–63^. The MAG data is one of the largest and most comprehensive bibliometrics data in the world, and a popular dataset for the science of science research. However, MAG was sunset by Microsoft at the end of 2021. Since then, there have also been several important efforts in the community to ensure the continuity of data and services. For example, there are mirror datasets^73^ available online for MAG, and the OpenAlex (https://openalex.org) initiative builds on the MAG data, and not only makes it open to all but also provides continuous updates^60^. While these efforts have minimized potential disruptions, the sunsetting of MAG has also accelerated the need to construct open data resources designed for research purposes. Indeed, large-scale systematic datasets for the science of science mostly come in the form of raw data, which requires further data pre-processing and filtering operations to extract fine-grained research data with high quality. It usually takes substantial efforts and expertise to clean the data, and many of these steps are often too detailed to document in publications, with researchers making their own analytical choices. It thus suggests that there is value in constructing an open data lake, which aims to continue to extend the usefulness of MAG, with substantial data pre-processing steps documented. Moreover, the data lake links together several disparate sources and pre-computed measures commonly used in the literature, serving as an open data resource for researchers interested in the quantitative studies of science and innovation.

Importantly, the curated data lake is not meant to be exhaustive; rather it represents an initial step toward a common data resource to which researchers across the community can collectively contribute. Indeed, as more data and measurements in the science of science become available, researchers can help to contribute to the continuous improvement of this data lake by adding new data, measurements, and linkages, thereby further increasing the utility of the data lake. For example, if a new paper reports a new measurement, the authors could publish a data file linking the new measurement with SciSciNet IDs, which would make it much easier for future researchers to build on their work.

## Methods

### Data selection and curation from MAG

The Microsoft Academic Graph (MAG) dataset^61–63^ covers a wide range of publication records, authors, institutions, and citation records among publications. MAG has a rich set of prominent features, including the application of advanced machine learning algorithms to classify fields of study in large-scale publication records, identify paper families, and disambiguate authors and affiliations. Here we use the edition released on December 6^th^, 2021 by MAG, in total covering 270,694,050 publication records.

The extensive nature of the MAG data highlights a common challenge. Indeed, using the raw data for research often requires substantial pre-processing and data-cleaning steps to arrive at a research-ready database. For example, one may need to perform a series of data selection and curation operations, including the selection of scientific publications with reliable sources, aggregation of family papers, and redistribution of citation and reference counts. After going through these steps, one may generate a curated publication data table, which serves as the primary scientific publication data table in SciSciNet (Table 3, “SciSciNet_Papers”). However, each of these steps requires us to make specific analytical choices, but given the detailed nature of these steps, the specific choices made through these steps have remained difficult to document through research publications.

**Table 3:** Data type for records of SciSciNet_Papers.

Index	Format	Short Description
PaperID	Integer	Unique MAG Paper ID of the paper.
DOI	String	Digital Object Identifier (DOI) of the paper.
DocType	String	Book, BookChapter, Conference, Dataset, Journal, Repository, Thesis, or NULL (unknown).
Year	Integer	Publication year of the paper.
Date	DateTime	Publication date of the paper formatted as YYYY-MM-DD.
JournalID	Integer	MAG Journal ID for published journal of the paper.
ConferenceSeriesID	Integer	MAG ConferenceSeries ID for published conference series of the paper.
Reference_Count	Integer	Total reference count of the paper.
Citation_Count	Integer	Total citation count of the paper.
C5	Integer	The number of citations 5 years after publication.
C10	Integer	The number of citations 10 years after publication.
Disruption	Float	Disruption score of the paper defined in Wu *et al*.^20^
Atyp_Median_Z	Float	Median Z-score of the paper defined in Uzzi *et al*.^47^
Atyp_10pct_Z	Float	10^th^ percentile Z-score of the paper defined in Uzzi *et al*.^47^
Atyp_Pairs	Integer	The number of journal pairs cite by the paper defined in Uzzi *et al*.^47^
WSB_mu	Float	Immediacy μ of the paper as introduced in WSB model^46^.
WSB_sigma	Float	Longevity σ of the paper as introduced in WSB model^46^.
WSB_Cinf	Integer	Ultimate impact of the paper predicted by WSB model^46^.
SB_B	Float	Beauty coefficient of the paper as introduced in Ke *et al*.^93^
SB_T	Integer	Awakening time of the paper as introduced in Ke *et al*.^93^
Team_Size	Integer	The number of researchers in the paper.
Institution_Count	Integer	The number of institutions in the paper.
Patent_Count	Integer	The number of citations by patents from USPTO and EPO.
Newsfeed_Count	Integer	The number of mentions by news from Newsfeed.
Tweet_Count	Integer	The number of mentions by tweets from Twitter.
NCT_Count	Integer	The number of citations by clinical trials from ClinicalTrials.gov.
NIH_Count	Integer	The number of supporting grants from NIH.
NSF_Count	Integer	The number of supporting grants from NSF.

Here we document in detail the various procedures we took in constructing the data lake. From the original publication data in MAG, we use MAG Paper ID as the primary key, and consider a subset of main attributes, including DOI (Digital Object Identifier), document type and publication year. As we are mainly interested in scientific publications within MAG, we first remove paper records whose document type is marked as patent. We also remove those with neither document type nor DOI information. Each scientific publication in the database may be represented by different entities (e.g., preprint and conference), indicated as a paper “family” in MAG. To avoid duplication, we aggregate all papers in the same family into one primary paper. We also do not include retracted papers in the primary paper table in SciSciNet. Instead, we include records of retracted papers and affiliated papers in paper families in another data table “SciSciNet_PaperDetails” (Table 8) linked to the primary paper table, recording information of DOIs, titles, original venue names, and original counts for citations and references in MAG. Following these steps, the primary data table “SciSciNet_Papers” contains 134,129,188 publication records with unique primary paper ids, including 90,764,813 journal papers, 4,629,342 books, 3,932,366 book chapters, 5,123,597 conference papers, 145,594 datasets, 3,083,949 repositories, 5,998,509 thesis papers, and 20,451,018 other papers with DOI information.

For consistency, we recalculate the citation and reference counts within the subset of 134 M primary papers, such that each citation or reference record is also included in this subset and can be found in “SciSciNet_PaperReferences” (Table 5). For papers in the same family, we aggregate their citations and references into the primary paper and drop duplicated citation pairs. Building on the updated citations, we recalculate the number of references and citations for each primary paper.

MAG also contains information of authors, institutions, and fields. While author disambiguation^58,74–79^ remains a major challenge, we adopt the author disambiguation method from MAG and create an author table, which offers a baseline for future studies of individual careers. We also supplement the author table with empirical name-gender associations to support gender research^80^, drawing from work by Van Buskirk *et al*.^80^; this allows us to build “SciSciNet_Authors_Gender” (Table 9) with 134,197,162 author records including their full names.

For fields, we use the fields of study records from MAG and focus on the records related to the selected primary papers (19 Level-0 fields and 292 Level-1 fields, Table 6). We incorporate this information into two tables, the “SciSciNet_PaperAuthorAffiliations” (Table 4) and “SciSciNet_PaperFields” (Table 7), with 413,869,501 and 277,494,994 records, respectively.

**Table 4:** Data type for records of SciSciNet_PaperAuthorAffiliations.

Index	Format	Short Description
PaperID	Integer	MAG Paper ID in the paper-author-affiliation record.
AuthorID	Integer	MAG Author ID in the paper-author-affiliation record.
AffiliationID	Integer	MAG Affiliation ID in the paper-author-affiliation record.
AuthorSequenceNumber	Integer	Original author sequence number starting with 1.

We further use the information of “PaperExtendedAttributes” table from MAG to construct high-quality linkages between MAG Paper ID and PubMed Identifier (PMID). We drop duplicate links by only keeping the MAG primary paper record (if one PMID was linked to multiple MAG Paper IDs) or the latest updated PubMed record (if one MAG Paper ID was linked to multiple PMIDs), obtaining 31,230,206 primary MAG Paper ID-PMID linkages (95.6% of the original records) to further support linkage with external sources.

Together, the resulting SciSciNet includes 134,129,188 publications (Table 3), 134,197,162 authors (Table 9), 26,998 institutions (Table 10), 49,066 journals (Tables 21), 4,551 conference series (Tables 22), 19 top-level fields of study, 292 subfields (Table 6), and the internal links between them, including 1,588,739,703 paper-references records (Table 5), 413,869,501 paper-author-affiliations records (Table 4), and 277,494,994 paper-fields records (Table 7).

### Linking publication data with external sources

While the main paper table captures citation relationships among scientific publications, there has been growing interest in studying how science interacts with other socioeconomic institutions^35,36,41,55,81,82^. Here, we further trace references of scientific publications in data sources that go beyond publication datasets, tracking the linkage between papers to their upstream funding supports and downstream uses in public domains. Specifically, here we link papers to the grants they acknowledge in NSF and NIH, as well as public uses of science by tracking references of scientific publications in patents, clinical trials, and news and social media.

#### NIH funding

The National Institutes of Health (NIH) is the largest public funder for biomedical research in the world. The recent decade has witnessed increasing interest in understanding the role of NIH funding for the advancement of biomedicine^81,82^ and its impact on individual career development^83,84^. NIH ExPORTER provides bulk NIH RePORTER (https://report.nih.gov/) data on research projects funded by the NIH and other major HHS operating divisions. The database also provides link tables (updated on May 16, 2021) that connects funded projects with resulting publications over the past four decades.

To construct the funded project-paper linkages between SciSciNet Paper ID and NIH Project Number, we use the PMID of MAG papers (from our previously curated “PaperExtendedAttributes” table based on MAG) as the intermediate key, matching more than 98.9% of the original NIH link table records to primary Paper ID in SciSciNet. After dropping duplicate records, we end up with a collection of 6,013,187 records (Table 11), linking 2,636,061 scientific papers (identified by primary MAG Paper IDs) to 379,014 NIH projects (identified by core NIH-funded project numbers).

#### NSF funding

Beyond biomedical research, the National Science Foundation (NSF) funds approximately 25% of all federally supported basic research conducted by the United States’ colleges and universities across virtually all fields of science and engineering. NSF provides downloadable information on research projects it has funded, including awardee, total award amount, investigator, and so forth, but no information on funded research publications. While Federal RePORTER offers downloadable files on NSF awards with links to supported publications (662,072 NSF award-publication records by 2019), it only covers a limited time period and has been retired by March 2022. To obtain a more comprehensive coverage of records linking NSF awards to supported papers, we crawl the webpages of all NSF awards to retrieve information on their resulting publications. In particular, we first created a comprehensive list of all NSF award numbers from https://www.nsf.gov/awardsearch/download.jsp. We then iterate over this list to download the entire webpage document of each NSF award (from the URL https://www.nsf.gov/awardsearch/showAward?AWD_ID = [Award number]), and use “Publications as a result of this research” column to identify scientific publications related to this award. We then extract paper titles and relevant information provided by using the Python library ElementTree to navigate and parse the webpage document structurally. We end up collecting 489,446 NSF awards since 1959 (Table 20), including linkages between 131,545 awards and 1,350,915 scientific publications.

To process information crawled from NSF.gov, which is presented as raw text strings, we design a text-based multi-level matching process to link NSF awards to SciSciNet scientific publications:

1. For records with DOI information in the raw texts of funded research publications, we perform an exact match with SciSciNet primary papers through DOI. If the DOI in an NSF publication record matched that of one primary paper, we create a linkage between the NSF Award Number and the primary Paper ID. We matched 458,463 records from NSF awards to SciSciNet primary papers, where each DOI appeared only once in the entire primary paper table, thus enabling association with a unique Paper ID (exact match). After dropping duplicates where the same DOI appears repeatedly in the same NSF award, we yield 350,611 records (26.0%) from NSF awards to SciSciNet primary papers.
2. To process the rest of the records, we then use the title information of each article for further matching. After extracting the title from NSF records and performing a standardization procedure (e.g., converting each letter into lowercase and removing punctuation marks, extra spaces, tabs, and newline characters), our exact matches between paper titles in the NSF award data and SciSciNet primary paper data yield 246,701 unique matches (18.3% in total) in this step.
3. We further develop a search engine for records that have not been matched in the preceding steps. Here we use Elasticsearch, a free and open search and analytics engine, to index detailed information (paper title, author, journal or conference name, and publication year) of all SciSciNet primary papers. We then feed raw texts of the crawled NSF publications into the system and obtain results with the top two highest scores associated with the indexed primary papers. Similar to a previous study^55^, we use scores of the second matched primary papers as a null model, and then identify the first matched primary paper as a match if its score is significantly higher than the right-tail cutoff of the second score distribution (*P* = 0.05). Following this procedure, we match the remaining 467,159 records (34.6%) from the two previous steps with significantly higher scores (Fig. 2a). Note that this procedure likely represents a conservative strategy that prioritizes precision over recall. Manually inspecting the rest of potential matchings, we find that those with large differences between the top two Z-scores (Fig. 2b) are also likely to be correct matches. To this end, we also include these heuristic links, together with the difference of their Z-scores, as fuzzy matching linkages between SciSciNet papers and NSF awards.

**Fig. 2:**
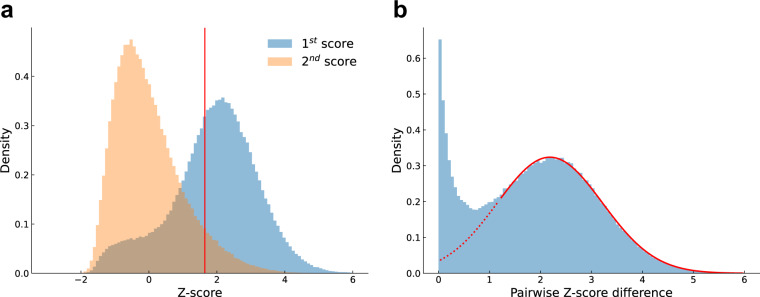
Matching NSF reference string to MAG records. (**a**) Distribution of Z-scores for papers matched in ElasticSearch with the first and second highest scores. The vertical red line denotes the right-tail cutoff of the second score distribution (*P* = 0.05). (**b**) Distribution of pairwise Z-score differences for papers matched in search engine but with the first score no higher than the right-tail cutoff of the second score distribution (*P* = 0.05).
4. We further supplement these matchings with information from Crossref data dump, an independent dataset that links publications to over 30 K funders including NSF. We collect all paper-grant pairs where the funder is identified as NSF. We then use the raw grant number from Crossref and link paper records between Crossref and SciSciNet using DOIs. We obtain 305,314 records after cleaning, including 196,509 SciSciNet primary papers with DOIs matching to 83,162 NSF awards.

By combining records collected from all these steps, we collect 1,130,641 unique linkages with high confidence levels and 178,877 additional possible linkages from fuzzy matches (Table 12). Together these links connect 148,148 NSF awards and 929,258 SciSciNet primary papers.

#### Patent citations to science

The process in which knowledge transfers from science to marketplace applications has received much attention in science and innovation literature^35,41,85–88^. The United States Patent and Trademark Office (USPTO) makes patenting activity data publicly accessible, with the PatentsView platform providing extensive metadata including as related to patent assignees, inventors, and lawyers, along with patents’ internal citations and full-text information. The European Patent Office (EPO) also provides open access to patent data containing rich attributes.

Building on recent advances in linking papers to patents^35,67,68^, Marx and Fuegi developed a large-scale dataset of over 40 M citations from USPTO and EPO patents to scientific publications in MAG. Using this corpus (Version v34 as of December 24, 2021), we merge 392 K patent citation received by affiliated MAG papers to their respective primary IDs in the same paper family. Dropping possible duplicate records with the same pair of primary Paper ID and Patent ID results in 38,740,313 paper-patent citation pairs between 2,360,587 patents from USPTO and EPO and 4,627,035 primary papers in SciSciNet (Table 15).

#### Clinical trials citations to science

Understanding bench-to-bed-side translation is essential for biomedical research^81,89^. ClinicalTrials.gov provides publicly available clinical study records covering 50 U.S. states and 220 countries, sourced from the U.S. National Library of Medicine. The Clinical Trials Transformation Initiative (CTTI) makes available clinical trials data through a database for Aggregate Analysis of ClinicalTrials.gov (AACT), an aggregated relational database helping researchers better study drugs, policies, publications, and other related items to clinical trials.

Overall, the data covers 686,524 records linking clinical trials to background or result papers (as of January 26th, 2022). We select 480,893 records with papers as reference background supporting clinical trials, of which 451,357 records contain 63,281 unique trials matching to 345,797 reference papers with PMIDs. Similar to the process of linking scientific publications to NIH-funded projects, we again establish linkages between SciSciNet primary Paper ID and NCT Number (National Clinical Trial Number) via PMID, aided by the curated “PaperExtendedAttributes” table as the intermediary. After standardizing the data format of the intermediate index PMID to merge publications and clinical trials, we obtain 438,220 paper-clinical linkages between 61,447 NCT clinical trials and 337,430 SciSciNet primary papers (Table 13).

#### News and social mentions of science

Understanding how science is mentioned in media has been another important research direction in the science of science community^44,90^. The Newsfeed mentions in Crossref Event Data link scientific papers in Crossref^59^ with DOIs to news articles or blog posts in RSS and Atom feeds, providing access to the latest scientific news mentions from multiple sources, including *Scientific American*, *The Guardian*, *Vox*, *The New York Times*, and others. Also, Twitter mentions in Crossref Event Data link scientific papers to tweets created by Twitter users, offering an opportunity to explore scientific mentions in Twitter.

We use the Crossref Event API to collect 947,160 records between 325,396 scientific publications and 387,578 webpages from news blogs or posts (from April 5^th^, 2017 to January 16^th^, 2022) and 59,593,281 records between 4,661,465 scientific publications and 58,099,519 tweets (from February 7^th^, 2017 to January 17^th^, 2022).

For both news media and social media mentions, we further link Crossref’s publication records to SciSciNet’s primary papers. To do so, we first normalize the DOI format of these data records and converted all alphabetic characters to lowercase. We use normalized DOI as the intermediate index, as detailed below:

For news media mentions, we construct linkages between primary Paper ID and Newsfeed Object ID (i.e., the webpage of news articles or blog posts) by inner joining normalized DOIs. We successfully link 899,323 records from scientific publications to news webpages in the Newsfeed list, accounting for 94.9% of the total records. The same news mention may be collected multiple times. After removing duplicate records, we end up with 595,241 records, linking 307,959 papers to 370,065 webpages from Newsfeed (Table 17).

Similarly, for social media mentions, we connect primary Paper IDs with Tweet IDs through inner joining normalized DOIs, yielding 56,121,135 records, more than 94% of the total records. After dropping duplicate records, we keep 55,846,550 records, linking 4,329,443 papers to 53,053,505 tweets (Table 16).

We also provide metadata of paper-news linkages, including the mention time and the detailed mention information in Newsfeed, to better support future research on this topic (Table 18). Similarly, we also offer the metadata of paper-tweet links, including the mention time and the original collected Tweet ID so that interested researchers can merge with further information from Twitter using the Tweet ID (Table 19).

#### Nobel Prize data from the dataset of publication records for Nobel laureates

We integrate a recent dataset by Li *et al*.^91^ in the data lake, containing the publication records of Nobel laureates in science from 1900 to 2016, including both Nobel prize-winning works and other papers produced in their careers. After mapping affiliated MAG Paper IDs to primary ones, we obtain 87,316 publication records of Nobel laureates in SciSciNet primary paper Table (20,434 in physics, 38,133 in chemistry, and 28,749 in physiology/medicine, Table 14).

### Calculation of commonly used measurements

Using the constructed dataset, we further calculate a range of commonly used measurements of scientific ideas, impacts, careers, and collaborations. Interested readers can find more details and validations of these measurements in the literature^15,19,20,46–48,92–98^.

#### Publication-level

##### The number of researchers and institutions in a scientific paper

Building on team science literature^19,27^, we calculate the number of authors and the number of institutions for each paper as recorded in our data lake. We group papers by primary Paper ID in the selected “SciSciNet_PaperAuthorAffiliations” table and aggregate the unique counts of Author IDs and Affiliation IDs as the number of researchers (team size) and institutions, respectively.

##### Five-year citations (*c*_5_), ten-year citations (*c*_10_), normalized citation (*c*_*f*_), and hit paper

The number of citations of a paper evolves over time^46,48,99,100^. Here we calculate *c*_5_ and *c*_10_, defined as the number of citations a paper received within 5 years and 10 years of publication, respectively. For the primary papers, we calculate *c*_5_ for all papers published up to 2016 (As the last version of MAG publication data is available until 2021) by counting the number of citation pairs with time difference less than or equal to 5 years. Similarly, we calculate *c*_10_ for all papers published up to 2011.

To compare citation counts across disciplines and time, Radicchi *et al*.^48^ proposed the relative citation indicator *c*_*f*_, as the total number of citations *c* divided by the average number of citations *c*_0_ in the same field and the same year. Here we calculate the normalized citation indicator for each categorized paper in both top-level fields and subfields, known as Level-0 fields (19 in total) and Level-1 fields (292 in total) categorized by MAG, respectively. Note that each paper may be associated with multiple fields, hence here we report calculated normalized citations for each paper-field pair in the “SciSciNet_PaperFields” data table.

Another citation-based measure widely used in the science of science literature^16,19,83^ is “hit papers”, defined as papers in the top 5% of citations within the same field and year. Similar to our calculation of *c*_*f*_, we use the same grouping by fields and years, and identify all papers with citations greater than the top 5% citation threshold. We also perform similar operations for the top 1% and top 10% hit papers.

##### Citation dynamics

A model developed by Wang, Song, and Barabási (the WSB model)^46^ captures the long-term citation dynamics of individual papers after incorporating three fundamental mechanisms, including preferential attachment, aging, and fitness. The model predicts the cumulative citations received by paper *i* at time *t* after publication: ${c}_{i}^{t}=m\left[{e}^{{{\rm{\lambda }}}_{i}\Phi \left(\frac{lnt-{{\rm{\mu }}}_{i}}{{{\rm{\sigma }}}_{i}}\right)}-1\right]$, where *Φ*(*x*) is the standard cumulative normal distribution of *x*, *m* captures the average number of references per paper, and μ_*i*_, σ_*i*_, and λ_*i*_ indicate the immediacy, longevity, and fitness parameters characterizing paper *i*, respectively.

We implement the WSB model with prior for papers published in the fields of math and physics. Following the method proposed by Shen *et al*.^92^, we adopt the Bayesian approach to calculate the conjugate prior, which follows a gamma distribution. The method allows us to better predict the long-term impact through the posterior estimation of *λ*_*i*_, while helping to avoid potential overfitting problems. Fitting this model to empirical data, we compute the immediacy *μ*_*i*_, the longevity *σ*_*i*_, and the ultimate impact ${c}_{{\rm{i}}}^{\infty }={\rm{m}}\left[{e}^{{{\rm{\lambda }}}_{i}}-1\right]$ for all math and physics papers with at least 10 citations within 10 years after publication (published no later than 2011). To facilitate research on citation dynamics across different fields^48^, we have also used the same procedure to fit the citation sequences for papers that have received at least 10 citations within 10 years across all fields of study from the 1960s to the 1990s.

##### Sleeping beauty coefficient

Sometimes it may take years or even decades for papers to gain attention from the scientific community, a phenomenon known as the “Sleeping Beauty” in science^93^. The sleeping beauty coefficient *B* is defined as ${\rm{B}}={\sum }_{t=0}^{{t}_{m}}\frac{\frac{{c}_{{t}_{m}}-{c}_{0}}{{t}_{m}}\cdot t+{c}_{0}-{c}_{t}}{{\rm{\max }}\left(1,{c}_{t}\right)}$, where the paper receives its maximum yearly citation ${c}_{{t}_{m}}$ in year *t*_*m*_ and *c*_0_ in the year of publication. Here we calculate the sleeping beauty coefficient from yearly citation records of a paper. We match the publication years for each citing-cited paper pair published in journals and then aggregate yearly citations since publication for each cited paper. Next, we group the “SciSciNet_PaperReferences” table by each cited paper and compute the coefficient *B*, along with the awakening time. As a result, we obtain 52,699,363 records with sleeping beauty coefficients for journal articles with at least one citation.

##### Novelty and conventionality

Research shows that the highest-impact papers in science tend to be grounded in exceptionally conventional combinations of prior work yet simultaneously feature an intrusion of atypical combinations^47^. Here following this work^47^, we calculate the novelty and conventionality score of each paper by computing the Z-score for each combination of journal pairs. We further calculate the distribution of journal pair Z-scores by traversing all possible duos of references cited by a particular paper. A paper’s median Z-score characterizes the median conventionality of the paper, whereas a paper’s 10^th^ percentile Z-score captures the tail novelty of the paper’s atypical combinations.

More specifically, we first use the information of publication years for each citing-cited paper pair both published in journals and shuffle the reference records within the citing-cited year group to generate 10 randomized citation networks, while controlling the naturally skewed citation distributions. We then traverse each focal paper published in the same year. We further aggregate the frequency of reference journal pairs for papers in the real citation network and 10 randomized citation networks, calculating the Z-score of each reference journal pair for papers published in the same year. Finally, for each focal paper, we obtain its 10^th^ percentile and median of the Z-scores distribution, yielding 44,143,650 publication records with novelty and conventionality measures for journal papers from 1950 to 2021.

##### Disruption score

Disruption index quantifies the extent to which a paper disrupts or develops the existing literature^20,51^. Disruption, or *D*, is calculated through citation networks. For a given paper, one can separate its future citations into two types. One type only cites the focal paper itself while ignoring all the references that the paper builds upon, and the other is to cite both the focal paper and its references. *D* is expressed as: ${\rm{D}}={{\rm{p}}}_{{\rm{i}}}-{{\rm{p}}}_{{\rm{j}}}=\frac{{n}_{i}-{n}_{j}}{{n}_{i}+{n}_{j}+{n}_{k}}$, where *n*_*i*_ is the number of subsequent works that only cite the focal paper, *n*_*j*_ is the number of subsequent works that cite both the focal paper and its references, and *n*_*k*_ is the number of subsequent works that cite the references of the focal paper only. Following this definition, we calculate the disruption scores for all the papers that have at least one forward and backward citation (48,581,274 in total).

##### The number of NSF and NIH supporting grants

For external linkages from scientific publications to upstream supporting funding sources, we calculate the number of NSF/NIH grants associated with each primary paper in SciSciNet.

##### The number of patent citations, Newsfeed mentions, Twitter mentions, and clinical trial citations

For external linkages from scientific publications to downstream public uses of science, we also calculate the number of citations each primary paper in SciSciNet received from domains that go beyond science, including patents from USPTO and EPO, news and social media mentions from Newsfeed and Twitter, and clinical trials from ClinicalTrials.gov.

#### Individual- and Institutional-level measures

##### Productivity

Scientific productivity is a widely used measure for quantifying individual careers^9,15^. Here we aggregate the unique primary Paper ID in SciSciNet, after grouping the records in the “SciSciNet_PaperAuthorAffiliations” data table by Author ID or Affiliation ID and calculate the number of publications produced by the same author or affiliation.

##### H-index

H-index is a popular metric to estimate a researcher’s career impact. The index of a scientist is *h*, if *h* of her papers have at least *h* citations and each of the remaining papers have less than *h* citations^94,101^. Here we compile the full publication list associated with each author, sort these papers by their total number of citations in descending order, and calculate the maximum value that satisfies the condition above as the H-index. By repeating the same procedure on each research institution, we also provide an institution-level H-index as well.

##### Scientific impact

Building on our *c*_10_ measure at the paper level, here we further calculate the average *c*_10_ (<*c*_10_>) for each author and affiliation, which offers a proxy to individual and institutional level scientific impact. Similarly, we calculate the average log*c*_10_ (<log*c*_10_>), which is closely related to the *Q* parameter^15^ of individual scientific impact.

Here we group by Author and Affiliation ID in the “PaperAuthorAffiliations” table, and then aggregate *c*_10_ and log*c*_10_ (pre-calculated at the paper level) of all papers published by the same id. Following previous works^15,16,102^, to avoid taking logarithm of zeros, we increase *c*_10_ by one when calculating the <log*c*_10_>.

##### Name-gender associations

The availability of big data also enables a range of studies focusing on gender disparities, ranging from scientific publications and careers^17,103–106^ to collaboration patterns^25,107^ and the effects of the pandemic on women scientists^45,108–110^. Here we apply the method from a recent statistical model^80^ to infer author gender based on their first names in the original author table. The method feeds unique author names into a cultural consensus model of name-gender associations incorporating 36 separate sources across over 150 countries. Note that for all the 134,197,162 authors, 23.26% of the authors (31,224,458) have only the first initials, which are excluded from the inference. By fine-tuning the annotated names from these data sources following the original method, we obtain 409,809 unique names with max uncertainty threshold set to 0.26 and 85% of the sample classified. Finally, we merge these name-gender inference records into the original SciSciNet_Authors table, resulting a SciSciNet_Authors_Gender table, which contains 86,286,037 authors with inferred probability that indicates a name belongs to an individual gendered female, denoted as P(gf), as well as the number of inference source datasets and empirical counts. Together, by combining new statistical models with our systematic authorship information, this new table provides name-gender information, useful in studying gender-related questions. It is important to note that such name-based gender inference algorithms, including the one used here as well as other popular tools such as *genderize.io*, have limitations and are necessarily imperfect. The limitations should be considered carefully when applying these methods^96^.

## Data Records

The data lake, SciSciNet, is freely available at Figshare^72^.

### Data structure

Table 2 presents the size and descriptions of these data files.

Table 3 contains information about “SciSciNet_Papers”, which is the data lake’s primary paper table, containing information on the primary scientific publications, including Paper ID, DOI, and others, along with the Journal ID or Conference Series ID, which can link papers to corresponding journals or conference series that take place regularly. The short description in each data field includes the corresponding explanation of that field.

Tables 4–22 include the data fields and corresponding descriptions of each data table. Each data field specified is clear from its index name. An ID of the data field in a data table can be linked, if this field has the same ID name as another field in another table. Further, the data link tables provide linkages from scientific publications to external socioeconomic institutions. For example, the paper with primary “PaperID” as “246319838”, which studied the hereditary spastic paraplegia^111^, lead to three core NIH project number “R01NS033645”, “R01NS036177”, and “R01NS038713” in the Table 11 “SciSciNet_Link_NIH”. We can not only extract detailed information and metrics of the paper in the data lake (e.g., title from Table 8 “SciSciNet_PaperDetails”, or citation counts from the primary paper Table 3 “SciSciNet_Papers”) but also obtain further information of the funded-projects, such as the total funding amount, from NIH RePORTER (https://report.nih.gov).

**Table 5:** Data type for records of SciSciNet_PaperReferences.

Index	Format	Short Description
Citing_PaperID	Integer	MAG Paper ID of the citing paper in the citation pair.
Cited_PaperID	Integer	MAG Paper ID of the cited paper in the citation pair.

**Table 6:** Data type for records of SciSciNet_Fields.

Index	Format	Short Description
FieldID	Integer	MAG Field ID of the field of study.
Field_Name	String	Original field name of the field of study.
Field_Type	String	Top or Sub. Top indicates the top-level field. Sub indicates the subfield.

**Table 7:** Data type for records of SciSciNet_PaperFields.

Index	Format	Short Description
PaperID	Integer	MAG Paper ID in the paper-field linkage record.
FieldID	Integer	MAG Field ID in the paper-field linkage record.
Hit_1pct	Integer	1 is hit paper with top 1% total citations within the same level field and the same year, and 0 is not.
Hit_5pct	Integer	1 is hit paper with top 5% total citations within the same level field and the same year, and 0 is not.
Hit_10pct	Integer	1 is hit paper with top 10% total citations within the same level field and the same year, and 0 is not.
C_f	Float	Normalized citation as defined by Radicchi *et al*.^48^

**Table 8:** Data type for records of SciSciNet_PaperDetails.

Index	Format	Short Description
PaperID	Integer	MAG Paper ID of the paper.
DOI	String	Digital Object Identifier (DOI) of the paper.
DocType	String	Book, BookChapter, Conference, Dataset, Journal, Repository, Thesis, or NULL (unknown).
PaperTitle	String	Title of the paper.
BookTitle	String	Book title of the paper.
Year	Integer	Publication year of the paper.
Date	DateTime	Publication date of the paper formatted as YYYY-MM-DD.
Publisher	String	Publisher name of the paper.
JournalID	Integer	MAG Journal ID for published journal of the paper.
ConferenceSeriesID	Integer	MAG ConferenceSeries ID for published conference series of the paper.
OriginalVenue	String	Original published venue name of the paper.
Volume	String	Volume of the paper.
Issue	String	Issue of the paper.
FirstPage	String	First page of the paper.
LastPage	String	Last page of the paper.
FamilyID	Integer	Primary MAG Paper ID of the paper in the same paper family.
RetractionType	String	“Retracted Publication”, “Retraction Notice”.
ReferenceCount	Integer	Reference count of the paper in MAG original papers data table.
CitationCount	Integer	Citation count of the paper in MAG original papers data table.

**Table 9:** Data type for records of SciSciNet_Authors_Gender.

Index	Format	Short Description
AuthorID	Integer	MAG Author ID of the author.
Author_Name	String	Original name of the author.
H-index	Integer	H-index of the author.
Productivity	Integer	Total number of publications of the author.
Average_C10	Float	Average *c*_10_ of the author.
Average_LogC10	Float	Average log*c*_10_ of the author.
Inference_Sources	Integer	The number of name-gender inference source datasets^80^.
Inference_Counts	Integer	The number of empirical count of humans with the first name and gendered label in the source datasets^80^.
P(gf)	Float	The probability that indicates to what extent a name belongs to an individual gendered female^80^.

**Table 10:** Data type for records of SciSciNet_Affiliations.

Index	Format	Short Description
AffiliationID	Integer	MAG Affiliation ID of the affiliation.
Affiliation_Name	String	Original name of the affiliation.
GridID	String	GRID (Global Research Identifier Database) ID of the affiliation.
Official_Page	String	Official webpage of the affiliation.
ISO3166Code	String	ISO 3166 two-letter country codes of the affiliation.
Latitude	Float	Latitude of the affiliation.
Longitude	Float	Longitude of the affiliation.
H-index	Integer	H-index of the affiliation.
Productivity	Integer	Total number of publications of the affiliation.
Average_C10	Float	Average c_10_ of the affiliation.
Average_LogC10	Float	Average log c_10_ of the affiliation.

**Table 11:** Data type for records of SciSciNet_Link_NIH.

Index	Format	Short Description
PaperID	Integer	MAG Paper ID.
NIH_Project_Number	String	NIH core project number.

**Table 12:** Data type for records of SciSciNet_Link_NSF.

Index	Format	Short Description
PaperID	Integer	MAG Paper ID.
NSF_Award_Number	String	NSF award number.
Type	String	“First” and “Crossref” are exact matches, and “Second” is fuzzy match. “Crossref” type is derived from Crossref funder-paper links.
Diff_ZScore	Float	The difference of Z-scores using heuristic method for the “Second” type.

**Table 13:** Data type for records of SciSciNet_Link_ClinicalTrials.

Index	Format	Short Description
PaperID	Integer	MAG Paper ID.
NCT_Number	String	National Clinical Trial number.

**Table 14:** Data type for records of SciSciNet_Link_NobelLaureates.

Index	Format	Short Description
PaperID	Integer	MAG Paper ID.
LaureateID	Integer	Nobel Laureate ID mentioned in Li *et al*.^91^
Type	Integer	1 is prize-wining paper, and 0 is not.

**Table 15:** Data type for records of SciSciNet_Link_Patents.

Index	Format	Short Description
PaperID	Integer	MAG Paper ID.
PatentID	String	Patent ID from the dataset by Marx and Fuegi’s work^67,68^.
Type	Integer	1 is from USPTO, and 0 is not.

**Table 16:** Data type for records of SciSciNet_Link_Twitter.

Index	Format	Short Description
PaperID	Integer	MAG Paper ID.
TweetID	Integer	Tweet ID.

**Table 17:** Data type for records of SciSciNet_Link_Newsfeed.

Index	Format	Short Description
PaperID	Integer	MAG Paper ID.
NewsfeedID	String	Newsfeed ID.

**Table 18:** Data type for records of SciSciNet_Newsfeed_Metadata.

Index	Format	Short Description
NewsfeedID	String	Newsfeed ID of the news article or blog post.
Occurred_Time	DateTime	Publication time of the news.
ObjectID	String	DOI object link of the mention.
Subject_Infomation	String	Detailed information of the subject news mention.

**Table 19:** Data type for records of SciSciNet_Twitter_Metadata.

Index	Format	Short Description
TweetID	Integer	Unique Tweet ID of the tweet.
Occurred_Time	DateTime	Publication time of the tweet.
ObjectID	String	DOI object link of the mention.
OriginalTweetID	String	Web link of the tweet.

**Table 20:** Data type for records of SciSciNet_NSF_Metadata.

Index	Format	Short Description
NSF_Award_Number	String	Unique NSF award number of the NSF award.
Title	String	Original title of the NSF award.
Publication_Research	String	Publications associated with the NSF award.
Date	DateTime	Date when the NSF award is signed by the NSF Grants Officer.

**Table 21:** Data type for records of SciSciNet_Journals.

Index	Format	Short Description
JournalID	Integer	MAG Journal ID of the journal.
Journal_Name	String	Original name of the journal.
ISSN	String	ISSN (International Standard Serial Number) of the journal.
Publisher	String	Original publisher of the journal.
Webpage	String	Original web link of the journal.

**Table 22:** Data type for records of SciSciNet_ConferenceSeries.

Index	Format	Short Description
ConferenceSeriesID	Integer	MAG ConferenceSeries ID of the conference series.
Abbr_Name	String	Abbreviated name of the conference series.
ConferenceSeries_Name	String	Original name of the conference series.

### Descriptive statistics

**Fig. 3:**
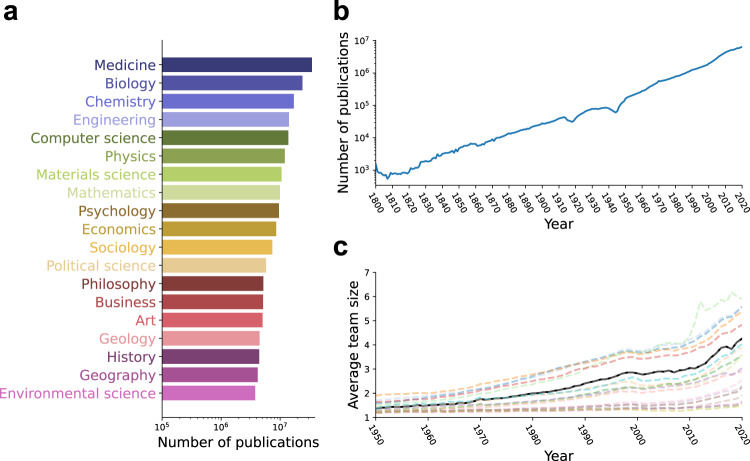
Summary statistics of scientific publications in SciSciNet. (**a**) The number of publications in 19 top-level fields. For clarity we aggregated the field classification into the top level (e.g., a paper is counted as a physics paper if it is associated with physics or any other subfields of physics). (**b**) The exponential growth of science over time. (**c**) Average team size by field from 1950 to 2020. The bold black line is for papers in all the 19 top-level fields. Each colored line indicates each of the 19 fields (color coded according to (a)).

Next, we present a set of descriptive statistics derived from the data lake. Figure 3a–c show the distribution of papers across 19 top-level fields, the exponential growth of scientific publications in SciSciNet over time, and the average team size of papers by field over time.

**Fig. 4:**
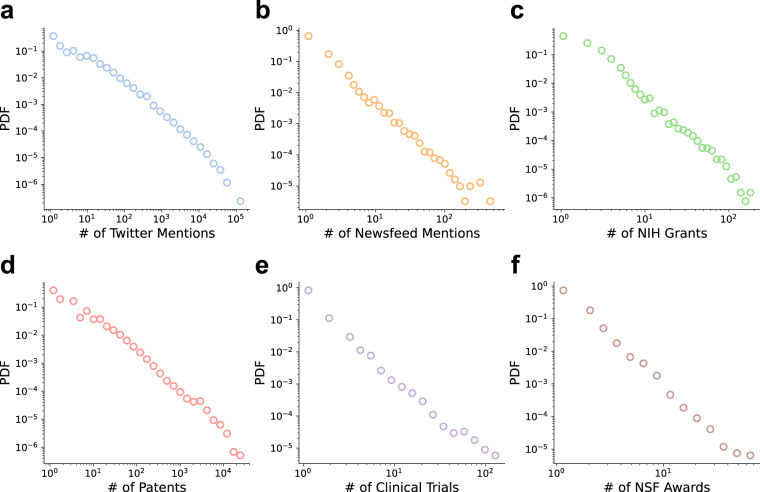
Linking scientific publications with socioeconomic institutions. Panels (**a,b** and **d,e**) show the distribution of paper-level downstream applications (**a**: Twitter mentions; **b**: Newsfeed mentions; **d**: Patents; **e**: Clinical trials). Panels (**c** and **f**) show the distribution of supporting scientific grants from NIH (**c**) and NSF (**f**).

Building on the external linkages we constructed, Fig. 4a–f show the distribution of paper-level upstream funding sources from NIH and NSF, and downstream applications and mentions of science, including USPTO/EPO patents, clinical trials, news mentions from Newsfeed, and social media mentions from Twitter.

**Fig. 5:**
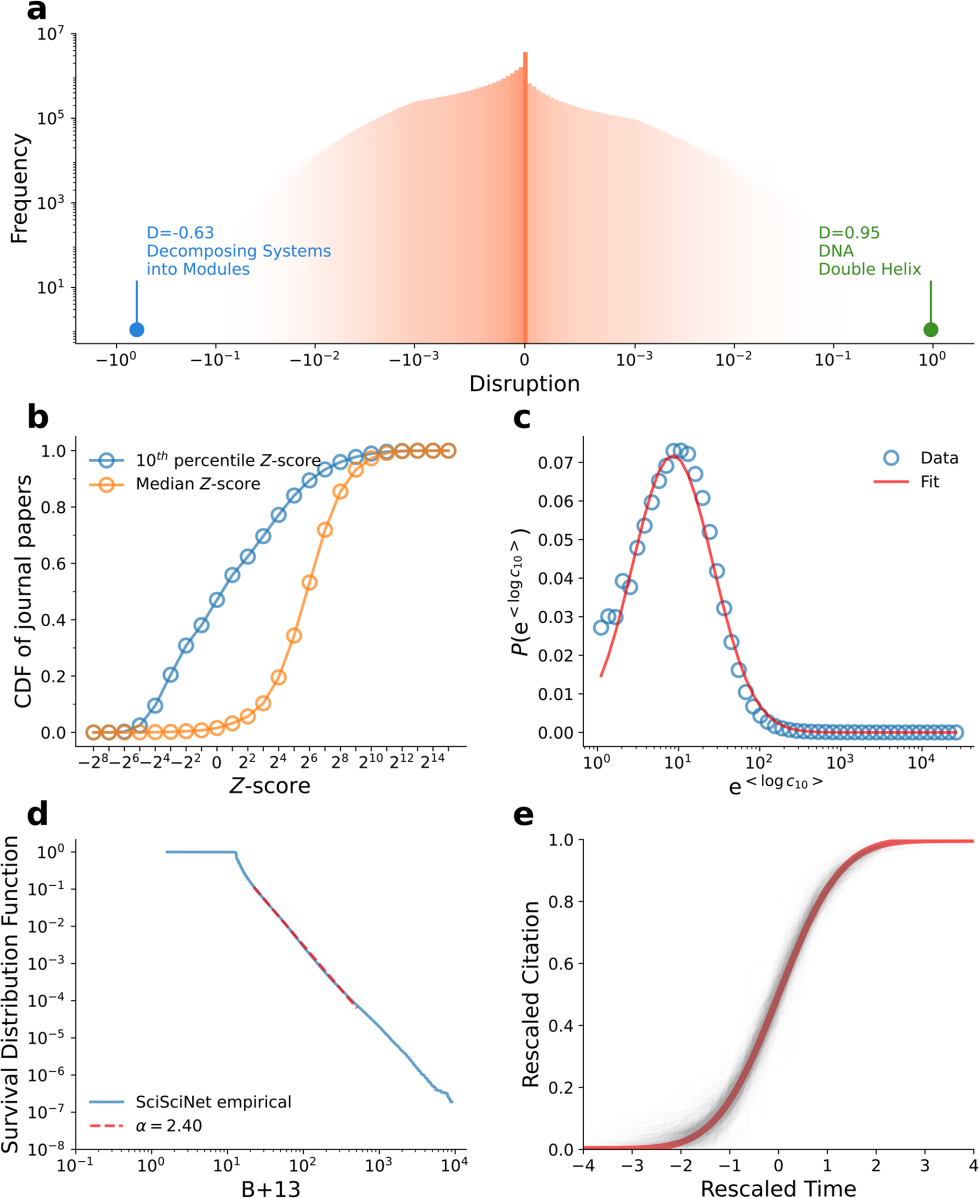
Commonly used metrics in SciSciNet. (**a**) The distribution of disruption score for 48,581,274 papers^20^ (50,000 bins in total). (**b**) Cumulative distribution function (CDF) of 44,143,650 journal papers’ 10^th^ percentile and median Z-scores^47^. (**c**) Distribution of ${e}^{{\rm{\langle }}log{c}_{\mathrm{10}}{\rm{\rangle }}}$ for scholars^15^ with at least 10 publications in SciSciNet. The red line corresponds to a log-normal fit with μ = 2.14 and *σ* = 1.14. (**d**) Survival distribution function of sleeping beauty coefficients^93^ for 52,699,363 papers, with a power-law fit: exponent *α* = 2.40. (**e**) Data collapse for a selected subset of papers with more than 30 citations within 30 years across journals in physics in the 1960s, based on WSB model^46^. The red line corresponds to the cumulative distribution function of the standard normal distribution.

Figure 5 presents the probability distributions of various commonly used metrics in the science of science using our data lake, which are broadly consistent with the original studies in the literature.

## Technical Validation

### Validation of publication and citation records

As we select the primary papers from the original MAG dataset, we have re-counted the citations and references within the subset of primary papers. To test the reliability of updated citation and reference counts in SciSciNet, here we compare the two versions (i.e., raw MAG counts and redistributed SciSciNet counts), by calculating the Spearman correlation coefficients for both citations and references. The Spearman correlation coefficients are 0.991 for citations and 0.994 for references, indicating that these metrics are highly correlated before and after the redistribution process.

We also examine the coverage of our publication data through a cross-validation with an external dataset, Dimensions^112^. By using DOI as a standardized identifier, we find that the two databases contain a similar number of papers, with 106,517,016 papers in Dimensions and 98,795,857 papers in SciSciNet associated with unique DOIs. We further compare the overlap of the two databases, finding the two data sources share a vast majority of papers in common (84,936,278 papers with common DOIs, accounting for 79.74% of Dimensions and 85.97% of SciSciNet).

Further, the citation information recorded by the two datasets appears highly consistent. Within the 84.9 M papers we matched with common DOIs, SciSciNet records a similar, yet slightly higher number of citations on average (16.75), compared with Dimensions (14.64). Our comparison also reveals a high degree of consistency in paper-level citation counts between the two independent corpora, with a Spearman correlation coefficient 0.946 and a concordance coefficient^98,113^ of 0.940. Together, these validations provide further support for the coverage of the data lake.

### Validation of external data linkages

We further perform additional cross-validation to understand the reliability of data linkages from scientific publications to external data sources. Here we focus more on the NSF-SciSciNet publications linkages we created from raw data collection to final data linkage. We also use the same approach to validate the NIH-SciSciNet publications linkages.

**Fig. 6:**
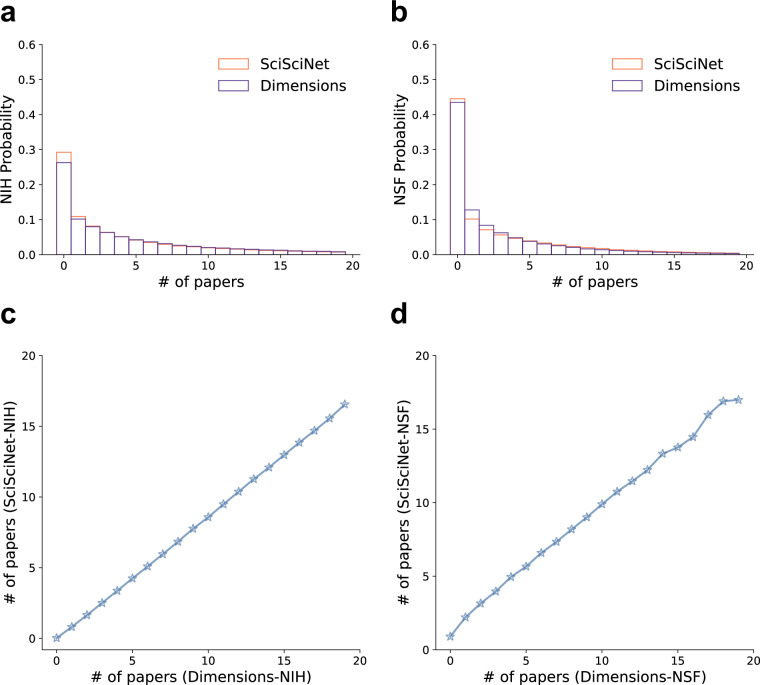
Validation of data linkages between SciSciNet and Dimensions. Panels (**a,b**), The distribution of number of papers matched to each NIH and NSF grant, respectively. Panels (**c,d**), The number of papers matched to each NIH and NSF grant, respectively. All panels are based on data in a 20-year period (2000–2020).

Here we compare the distribution and coverage of paper-grants linkages between SciSciNet and Dimensions—one of the state-of-the-art commercial databases in publication-grant linkages^112^. Figure 6a,b present the distribution of the number of papers matched to each NSF award and NIH grant, showing that our open-source approach offers a comparable degree of coverage. We further perform individual grant level analysis, by comparing the number of papers matched to each grant reported by the two sources (Fig. 6c,d), again finding high degrees of consistency (Spearman correlation coefficient: 0.973 for NIH grants and 0.714 for NSF grants).

We further calculate the confusion matrices of linkage from SciSciNet and Dimensions. By connecting the two datasets through paper DOIs and NSF/NIH grant project numbers, we compare their overlaps and differences in grant-paper pairs. For NSF, the confusion matrix is shown in Table 23. The two datasets provide a similar level of coverage, with Dimensions containing 670,770 pairs and SciSciNet containing 632,568 pairs. 78.9% pairs in Dimensions (and 83.7% pairs in SciSciNet) can be found in the other dataset, documenting a high degree of consistency between the two sources. While there are data links contained in Dimensions that are not in SciSciNet, we also find that there exists a similar amount of data records in SciSciNet but not in Dimensions. Table 24 shows the confusion matrix of NIH grant-paper pairs between the two datasets. Again, the two datasets share a vast majority of grant-paper pairs in common, and 95.3% pairs in Dimensions (and 99.7% pairs in SciSciNet) can also be found in the other dataset. These validations further support the overall quality and coverage of data linkages in SciSciNet.

**Table 23:** Confusion table of pairs of NSF grant-paper with DOI between SciSciNet and Dimensions.

NSF grant-paper pairs	In SciSciNet	Not in SciSciNet
In Dimensions	529,382	141,388
Not in dimensions	103,186	\

**Table 24:** Confusion table of pairs of NIH grant-paper with DOI between SciSciNet and Dimensions.

NIH grant-paper pairs	In SciSciNet	Not in SciSciNet
In Dimensions	5,356,652	264,119
Not in dimensions	15,157	\

### Validation of calculations of commonly used measurements

We also seek to validate the calculated metrics included in SciSciNet. In addition to manual inspection of independent data samples during data processing, along with presenting the corresponding distributions of indicators in the Descriptive statistics section, which capture general patterns, we further double-check the calculation results of these popular measurements in SciSciNet by reproducing canonical results in the science of science under a series of standardized and transparent processes.

#### Disruption

**Fig. 7:**
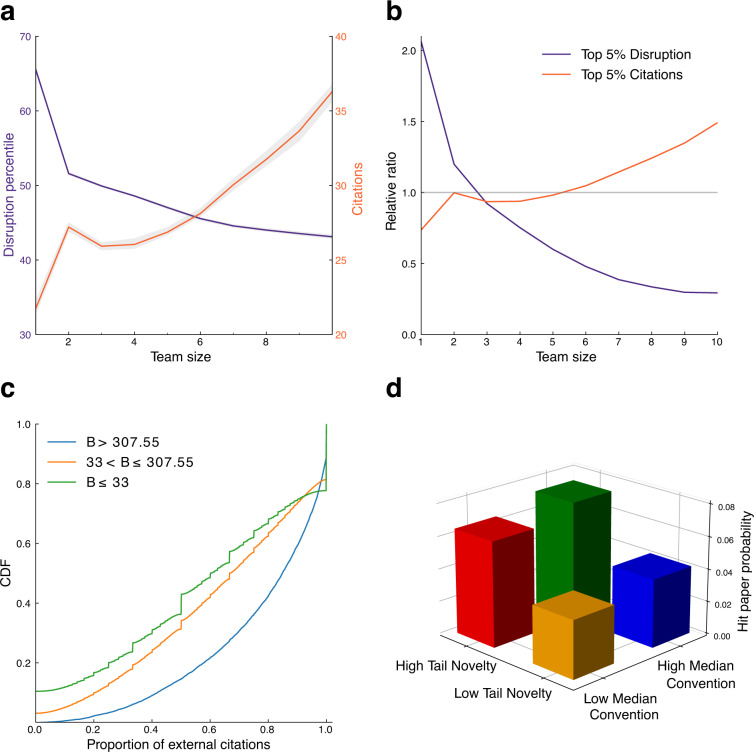
Calculating commonly used measurements in the science of science literature. (**a,b**), Small teams disrupt while large teams develop in SciSciNet. (**c**), The cumulative distribution functions (CDFs) of proportion of external citations for papers with high (top 10,000, B > 307.55), medium (from 10,001^st^ to top 2% SBs, 33< B < = 307.55); and low (B < = 33) sleeping beauty indexes. (**d**), The probability of a 5% hit paper, conditional on novelty and conventionality for all journal articles in SciSciNet from 1950 to 2000.

For disruption scores, we plot the median disruption percentile and average citations on different team sizes for 48,581,274 publications with at least one citation and reference record in SciSciNet. As shown in Fig. 7a, when team size increases, the disruption percentile decreases while the average citations increase, which is consistent with the empirical findings that small teams disrupt whereas large teams develop^20^. In addition, the probability of being among the top 5% disruptive publications is negatively correlated with the team size, while the probability of being among the most impactful publications increases is positively correlated with the team size (Fig. 7b). These results demonstrate the consistency with results obtained in the literature.

#### Novelty and conventionality

The combinations of conventional wisdom and atypical knowledge tend to predict a higher citation impact^47^. Here we repeat the original analysis by categorizing papers based on (1) median conventionality: whether the median score of a paper is in the upper half and (2) tail novelty: whether the paper is within the top 10^th^ percentile of novelty score. We then identified hit papers (within the subset of our analysis), defined as papers rank in the top 5% of ten-year citations within the same top-level field and year. The four quadrants in Fig. 7d suggest that papers with high median conventionality and high tail novelty present a higher hit rate of 7.32%, within the selection of SciSciNet papers published from 1950 to 2000. Also, papers with high median conventionality but low tail novelty show a hit rate of 4.18%, roughly similar to the baseline rate of 5%, while those with low median conventionality but high tail novelty display a hit rate of 6.48%. Meanwhile, papers with both low median conventionality and low tail novelty exhibit a hit rate of 3.55%. These results are broadly consistent with the canonical results reported in^47^.

#### WSB model

In Fig. 5e, we select 36,802 physics papers published in the 1960s with more than 30 citations within 30 years of publication. By rescaling their citation dynamics using the fitted parameters, we find a remarkable collapse of rescaled citation dynamics which appears robust across fields and decades. We further validate the predictive power of the model with prior based on Shen *et al*.^92^, by calculating the out-of-sample prediction accuracy. We find that with a training period of 15 years, the predictive accuracy (defined as a strict absolute tolerance threshold of 0.1) stays above 0.65 for 10 years after the training period, and the Mean Absolute Percentage Error (MAPE) is less than 0.1. The MAPE stays less than 0.15 for 20 years after the training period.

#### Sleeping beauty

We first fit the distribution of the sleeping beauty coefficients in SciSciNet (Fig. 5d) to a power-law form using maximum likelihood estimation^114^, obtaining a power-law exponent *α* = 2.40 and minimum value *B*_*m*_ = 23.59. By using fine-grained subfield information provided by MAG, we further calculate the proportion of external citations. Consistent with the original study^93^, we find that papers with high *B* scores are more likely to have a higher proportion of external citations from other fields (Fig. 7c).

## Usage Notes

Note that, recognizing the recent surge of interest in quantitative understanding of science^95,97,98,115,116^, the measurements currently covered in the data lake are not meant to be comprehensive; rather they serve as examples to illustrate how researchers from the broader community can collectively contribute and enrich the data lake. There are also limitations of the data lake that readers should keep in mind when using the data lake. For example, our grant-publication linkage is focused on scientific papers supported by NSF and NIH; patent-publication linkage is limited to citations from USPTO and EPO patents; clinical trial-publication linkage is derived from clinitrials.gov (where the geographical distribution may be heterogenous across countries, Table 25); and media-publication linkage is based on sources tracked by Crossref. Further, while our data linkages are based on state-of-the-art methods of data extraction and cleaning, as with any matching, the methods are necessarily imperfect and may be further improved through integration with complementary commercial products such as Altmetric and Dimensions. Finally, our data inherently represents a static snapshot, drawing primarily from the final edition of MAG (Dec 2021 version). While this snapshot is already sufficient in answering many of the research questions that arise in the field, future work may engage in continuous improvement and update of the data lake to maximize its potential.

**Table 25:** Top 10 countries ranked by the number of clinical trials (left) and the number of clinical trials linked to scientific papers (right).

Country	# Clinical Trials	Country	# Clinical Trials linked to papers
United States	153,632	United States	22,358
France	31,328	Canada	3,666
Canada	26,036	China	3,099
China	24,095	France	3,036
Germany	23,669	Italy	2,907
United Kingdom	22,304	Germany	2,703
Spain	17,454	United Kingdom	2,554
Italy	17,163	Spain	2,351
Korea, Republic of	13,213	Turkey	1,712
Belgium	12,182	Netherlands	1,456

Overall, this data lake serves as an initial step for serving the community in studying publications, funding, and broader impact. At the same time, there are also several promising directions for future work expanding the present effort. For example, the rapid development in natural language processing (NLP) models and techniques, accompanied by the increasing availability of text information from scientific articles, offers new opportunities to collect and curate more detailed content information. For example, one can link SciSciNet to other sources such as OpenAlex or Semantic Scholar to analyze large-scale data of abstract, full-text, or text-based embeddings. Such efforts will not only enrich the metadata associated with each paper, but also enable more precise identification and linkage of bio/chemical entities studied in these papers^117^. Further, although platforms like MAG have implemented advanced algorithms for name disambiguation and topic/field classification at scale, these algorithms are inherently imperfect and not necessarily consistent across datasets, hence it is essential to further validate and improve the accuracy of name disambiguation and topic classifications^118^. Related, in this paper we primarily focus on paper-level linkages across different datasets. Using these linkages as intermediary information, one can further construct and enrich individual-level profiles, allowing us to combine professional information (e.g., education background, grants, publications, and other broad impact) of researchers with important demographic dimensions (e.g., gender, age, race, and ethnicity). Finally, the data lake could contribute to an ecosystem for the collective community of the science of science. For example, there are synergies with the development of related programming packages, such as pySciSci^119^. By making the data lake fully open, we also hope it inspires other researchers to contribute to the data lake and enrich its coverage. For example, when a research team publishes a new measure, they could put out a data file that computes their measure based on SciSciNet, effectively adding a new column to the data lake. Lastly, science forms a complex social system and often offers an insightful lens to examine broader social science questions, suggesting that the SciSciNet may see greater utility by benefiting adjacent fields such as computational social science^120,121^, network science^122,123^, complex systems^124^, and more^125^.

## Supplementary information

SUPPLEMENTARY INFORMATION

## Data Availability

The source code for data selection and curation, data linkage, and metrics calculation is available at https://github.com/kellogg-cssi/SciSciNet.
