# Supplementary material for: SciSciNet: A large-scale open data lake for the science of science research
Source: Sci Data. 2023 Jun 1;10:315. doi: 10.1038/s41597-023-02198-9 (PMC10235093; doi:10.1038/s41597-023-02198-9)
Supplement: Supplementary file 1 — SUPPLEMENTARY INFORMATION [file 41597_2023_2198_MOESM1_ESM.docx]

**Supplementary Information**

**SciSciNet: A large-scale open data lake for the science of science research**

Zihang Lin, Yian Yin, Lu Liu, Dashun Wang*

*Corresponding author: [dashun.wang@northwestern.edu](mailto:dashun.wang@northwestern.edu)

**Table of Contents**

**Table S1** Detailed summary of major data sources commonly used in the science of science literature.

**Supplementary Table**

| **Data source** | **Data Coverage** | **Highlights** | **Access** | |
| --- | --- | --- | --- | --- |
|  |  |  | **API** | **Data**  **dump** |
| [Crossref](https://www.crossref.org/) | 120M+ records of publication metadata with DOIs registered in Crossref. | Event data linking papers to their online references (e.g., mainstream and social media). | ✓ | ✓ |
| [OpenAlex](https://openalex.org/) | 210M+ publication records inherited from MAG and their own subsequent collections. | Metadata of papers, disambiguated authors, venues, institutions, concepts, and linkages to other databases. | ✓ | ✓ |
| [Dimensions](https://www.dimensions.ai/) | 126M+ publications, 6M+ grants, 11M+ datasets, 743K+ policy documents, 693K+ clinical trials, and 144M+ patents. | Linkages between publications, grants, datasets, policy documents, clinical trials, and patents. | – | – |
| [Overton](https://www.overton.io/) | 4.7M+ policy documents worldwide. | 10.8M+ policy-science citations. | – | – |
| [OpenCitations](https://opencitations.net/) | 71M+ publications. | 1.2B+ DOI-DOI open citation links. | ✓ | ✓ |
| [AMiner](https://www.aminer.org/) | 130M+ researchers, 185M+ publications, and 8M+ concepts. | Rich information generated by data mining methods (e.g., the Science Knowledge Graph). | ✓ | ✓ |
| [CiteSeerX](https://citeseerx.ist.psu.edu/index) | 10M+ full-text publications with metadata, 32M+ authors, and 240M+ citations. | One of the earliest digital library search engines, complete open-source software (where possible). | ✓ | – |
| [ORCID](https://orcid.org/) | 13M+ researchers with ORCID identifiers. | Information on funding, affiliations, works and peer review. | ✓ | ✓ |
| [ROR](https://ror.org/) | 100K+ organizations with unique identifiers in the world. | Originally seeded by GRID, providing address, name variants, and other metadata information. | ✓ | ✓ |
| [Retraction Watch](https://retractionwatch.com/) | 32K+ publication retractions. | Metadata of retracted papers and reasons for retraction. | ✗ | – |
| [Semantic Scholar](https://www.semanticscholar.org/) | 204M+ papers with AI-powered search engine and advanced NLP techniques. | Large-scale text data (paper abstracts and full text for open-access papers) and derived paper-level embeddings. | ✓ | – |
| [Web of Science](https://www.webofknowledge.com/) | 82M+ publications records in the Web of Science Core Collection. | Curated by a team of expert in-house editors, served as the basis for products such as Journal Citation Reports and Highly Cited Researchers. | – | – |
| [PubMed](https://pubmed.ncbi.nlm.nih.gov/) | 33M+ biomedical literature with unique PubMed identifiers (PMID). | Linkages to funded projects, clinical trials, and Medical Subject Headings (MeSH) terms. | ✓ | ✓ |
| [NIH RePORTER](https://report.nih.gov/) | 2.59M+ NIH-funded projects. | Detailed information on NIH-funded projects, with linkages to publications, patents, and clinical studies. | ✓ | ✓ |
| [NSF Awards](https://www.nsf.gov/awardsearch/) | 489k+ NSF-funded projects. | Detailed information on NSF-funded projects, along with linkages to publications. | ✓ | ✓ |
| [Clinical Trials](https://clinicaltrials.gov/) | 403K+ clinical studies worldwide. | Information on clinical studies and linkages to references. | ✓ | ✓ |
| [USPTO PatentsView](https://patentsview.org/) | 7.9M+ patents granted by USPTO and 5.9M+ pre-grant applications. | Information on internal citations, patent classes, and disambiguated assignees and inventors. | ✓ | ✓ |
| [Reliance on Science in Patenting](https://zenodo.org/record/5803985) | 40M+ patent-article citation records extracted from 16M+ USPTO and EPO patents. | Both front-page and in-text patent-to-article citations with hand-tuned and machine learning techniques. | ✗ | ✓ |
| [Publication records of Nobel laureates](https://doi.org/10.7910/DVN/6NJ5RN) | 545 Nobel laureates, with 873 prize-winning papers and 93K+ publication records. | Extensive coverage of Nobel laureates including their full publication records and prize-winning papers. | ✗ | ✓ |
| [Altmetric](https://www.altmetric.com/) | 191M+ mentions of 35M+ research outputs. | Mentions from multiple sources, such as Wikipedia, policy documents, and mainstream and social media. | ✓ | – |
| [CORE](https://core.ac.uk/) | 207M+ papers. | Metadata and full-text information of 87M+ papers. | ✓ | ✓ |
| [Unpaywall](https://unpaywall.org/) | 31M+ articles. | Publication metadata and open-access related information. | ✓ | ✓ |
| [DOAJ](https://doaj.org/) | 17K+ journals, 7M+ articles in the directory of open access journals. | A community-curated directory providing open-access, peer-reviewed journals and papers. | ✓ | ✓ |
| [OpenAIRE](https://www.openaire.eu/)  [Research Graph](https://www.openaire.eu/) | 3M+ projects and 172M+ research products. | Metadata and links between scientific products, organizations, funded projects, etc. from 70K+ sources. | ✓ | ✓ |
| [Faculty Opinions with Gender](https://data.ub.uni-muenchen.de/288/) | 120K+ focal authors with inferred genders in 38K focal papers, with 335K subsequently cited papers. | Metadata of authors from Faculty Opinions with classified gender and papers from Faculty Opinions and Web of Science. | – | ✓ |
| [Scopus](https://www.scopus.com/) | 82M+ documents, selected by an independent review board of experts. | A large-scale database of interconnected publications (internal citations), researchers, and institutions. | – | – |
| [Lens](https://www.lens.org/) | 245M+ scholarly works, 137M+ patents, and 371M+ biological sequences. | 1.7B+ scholarly-work internal citations, 330M+ patent internal citations, and 4M+ patent- paper citations. | – | – |
| [Springer Nature SciGraph](https://www.springernature.com/gp/researchers/scigraph) | 9M+ articles, 301K+ books, 4M+ chapters, 102K+ organizations, and 8M+ persons. | 1.5B+ triples across research landscape, including publications, funders, and affiliations. | ✓ | ✓ |
| [Google Scholar](https://scholar.google.com/) | Large-scale dataset of publications, citations, and scholar profiles indexed by Google. | Scholar profile combining author disambiguation algorithms and additional information from scholars. | ✗ | ✗ |

**Table S1. Detailed summary of major data sources commonly used in the science of science literature.** ✓: publicly available, –: available upon application or subscription, ✗: not available to the best of our knowledge.
